# Psychological, Cultural, and Linguistic Barriers to Presenting New Ideas

**DOI:** 10.31662/jmaj.2025-0342

**Published:** 2025-09-12

**Authors:** Shunsuke Koga

**Affiliations:** 1 Department of Pathology and Laboratory Medicine, Hospital of the University of Pennsylvania, Philadelphia, PA, USA

**Keywords:** academic writing, letter, medical trainees, linguistic barriers, artificial intelligence, large language models

## Dear Editors,

I read with interest the recent article by Saeki emphasizing the importance of presenting new ideas and opinions in academic journals, particularly through Letters to the Editor and Opinion pieces ^(1)^. As a Japanese physician currently in residency training in the United States, I strongly support his suggestions. Here, I would like to share my personal perspective concerning psychological, cultural, and linguistic barriers that may discourage Japanese medical trainees from submitting these types of manuscripts.

As Saeki notes, letters and opinion pieces are easier to write than original research articles, as these formats typically do not require Institutional Review Board approval, research funding, or data collection. Nevertheless, psychological and cultural barriers still prevent Japanese trainees from submitting such manuscripts independently.

One major barrier is psychological and cultural, significantly impacting Japanese trainees’ willingness to independently submit letters or opinion pieces. Japan’s traditional hierarchical academic system (“ikyoku”) can implicitly discourage junior physicians from independently writing or submitting manuscripts without senior co-authors, as such actions might be perceived as professionally inappropriate or disrespectful. Furthermore, Japanese physicians commonly prefer anonymous accounts on social media platforms, even when discussing professional and scientifically valid topics, in sharp contrast to the common practice of openly using real names in the United States. This cultural preference for anonymity might create an additional psychological barrier when trainees consider publicly expressing their perspectives in academic journals.

Another critical barrier is linguistic insecurity, as researchers from non-English-speaking countries, including Japan, commonly face significant challenges due to the dominance of English in academic publishing ^(2)^. However, recent advancements in artificial intelligence (AI), notably large language models, offer promising tools for overcoming these linguistic barriers ^(3)^. A recent study has provided empirical evidence showing substantial improvement in English academic writing proficiency among non-native English-speaking medical students through the use of ChatGPT ^(4)^. Nevertheless, integrating explicit education on the ethical and practical application of AI tools into medical curricula is essential to fully realize their potential in facilitating scientific writing ^(5)^.

In summary, addressing psychological, cultural, and linguistic barriers is essential to promote manuscript submissions among Japanese medical trainees. Practical guidance, such as the checklist provided by Saeki ^(1)^ and effective use of AI-based writing assistance tools will enhance trainees’ confidence. Ultimately, these efforts will amplify contributions from Japan, increasing diversity within international academic discussions.

## Article Information

### Conflicts of Interest

None

### Author Contributions

Shunsuke Koga: Conceptualization, Manuscript drafting.

### Ethical Approval

Not applicable.
